# Highly-multiplexed and efficient long-amplicon PacBio and Nanopore sequencing of hundreds of full mitochondrial genomes

**DOI:** 10.1186/s12864-023-09277-6

**Published:** 2023-05-02

**Authors:** Benjamin R. Karin, Selene Arellano, Laura Wang, Kayla Walzer, Aaron Pomerantz, Juan Manuel Vasquez, Kamalakar Chatla, Peter H. Sudmant, Bryan H. Bach, Lydia L. Smith, Jimmy A. McGuire

**Affiliations:** 1 Department of Integrative Biology, Valley Life Sciences Building, University of California, Berkeley, CA 94708 USA; 2 Museum of Vertebrate Zoology, University of California, Berkeley, CA USA; 3 Center for Computational Biology, University of California, Berkeley, CA USA; 4 Department of Environmental Science, Policy, and Management, University of California, Berkeley, CA USA; 5 Innovative Genomics Institute, University of California, Berkeley, CA USA

**Keywords:** mtDNA, DNA barcoding, MinION, LongAmp, Third generation sequencing, Long read sequencing, Plasmid

## Abstract

**Background:**

Mitochondrial genome sequences have become critical to the study of biodiversity. Genome skimming and other short-read based methods are the most common approaches, but they are not well-suited to scale up to multiplexing hundreds of samples. Here, we report on a new approach to sequence hundreds to thousands of complete mitochondrial genomes in parallel using long-amplicon sequencing. We amplified the mitochondrial genome of 677 specimens in two partially overlapping amplicons and implemented an asymmetric PCR-based indexing approach to multiplex 1,159 long amplicons together on a single PacBio SMRT Sequel II cell. We also tested this method on Oxford Nanopore Technologies (ONT) MinION R9.4 to assess if this method could be applied to other long-read technologies. We implemented several optimizations that make this method significantly more efficient than alternative mitochondrial genome sequencing methods.

**Results:**

With the PacBio sequencing data we recovered at least one of the two fragments for 96% of samples (~ 80–90%) with mean coverage ~ 1,500x. The ONT data recovered less than 50% of input fragments likely due to low throughput and the design of the Barcoded Universal Primers which were optimized for PacBio sequencing. We compared a single mitochondrial gene alignment to half and full mitochondrial genomes and found, as expected, increased tree support with longer alignments, though whole mitochondrial genomes were not significantly better than half mitochondrial genomes.

**Conclusions:**

This method can effectively capture thousands of long amplicons in a single run and be used to build more robust phylogenies quickly and effectively. We provide several recommendations for future users depending on the evolutionary scale of their system. A natural extension of this method is to collect multi-locus datasets consisting of mitochondrial genomes and several long nuclear loci at once.

**Supplementary Information:**

The online version contains supplementary material available at 10.1186/s12864-023-09277-6.

## Introduction

Mitochondrial DNA has long been used to study biodiversity as it contains genes that can be used to build phylogenetic trees across Eukaryotes, contains abundant phylogenetic signal due to its rapid evolutionary rate and reduced effective population size, and encodes genes with clear functional roles in cellular respiration. Single mitochondrial genes are commonly used for DNA barcoding, phylogenetic trees, and many other uses, though the amount of sequence data that could be easily obtained was previously limited by sequencing technology [1]. Whole mitochondrial genomes (mitogenomes) are preferable to single mitochondrial genes for several reasons, including increased phylogenetic information content, alignment with differing genetic resources from previous research, and opening the ability to more thoroughly study mitochondrial adaptation and bioenergetics [2], gene rearrangement and structural variation [3], and Dobzhansky-Muller incompatibilities [4]. Mitogenome sequencing has become commonplace in recent years as costs continue to decline [5]. We sought to leverage new technology to create a simple and cost-effective protocol for generating large numbers of mitogenomes in parallel by applying long-amplicon sequencing methods. As mitogenomes become easier to obtain, they will be used more often as a phylogenetic barcoding marker, and as more are sequenced and deposited on online databases they will lead to the study of mitogenome variation across biodiversity. Applying these methods to portable long-read sequencing technology will enable complete lab work and data processing to be conducted in field settings and in locations with limited laboratory resources [6].

The most common approach for Illumina-based mitogenome sequencing is genome-skimming, or simply sequencing whole genome samples at enough depth to cover the mitogenome, which can correspond to 1–2% or more of reads [7, 8]. This method is effective yet inefficient in that it produces substantial off-target reads and can lead to chimeras from NuMt sequences [11]. Other possible methods to enrich the mitogenome include hybrid-capture [9] and enzymatic depletion of linear DNA [10], but these methods still require adapter ligation which imposes significant financial costs especially when scaling up the number of samples (see additional file 1). The primary way to avoid the adapter ligation step during short-read sequencing preparation is by using PCR-based indexing (also known as Illumina amplicon sequencing), as is commonly applied to 16 S rRNA metabarcoding [12] and DNA-barcoding approaches [11, 13, 14], but this carries other downsides if applied to the whole mitogenome as it is limited by the read-length available with short-read sequencing. Specifically, paired-end fragments are only 300 bp for HiSeq and NovaSeq and 600 bp for MiSeq [15], requiring in most cases for amplicons to be shorter than this (and even shorter still after accounting for primers, adapters, and indexes). The most efficient method to overcome the fixed per-sample cost of adapter ligation in short-read sequencing of the entire mitogenome, as far as we know, is to employ a tiled multiplex PCR strategy using short overlapping amplicons [16, 17, but also see 18]. These amplicons can then be transformed into libraries using indexing PCR, thus eliminating the need for adapter ligation. This method, however, will be difficult to optimize with so many primer pairs and to work effectively across broader evolutionary scales.

Recent advancements in the accuracy of long-read sequencing technologies, such as Oxford Nanopore Technologies (ONT) and Pacific Biosciences Sequel II (PacBio), have prompted the use of parallel sequencing for mtDNA and other amplicons in biodiversity science. These technologies offer efficient and cost-effective options for large-scale sequencing. For example, Hebert et al. [19] utilized PCR-based, asymmetric indexing to sequence more than 9,000 658 bp mitochondrial COI amplicons from a single PacBio SMRT cell at a low cost per sample [also see 20]. Other researchers have expanded on this amplicon sequencing method for nuclear and bacterial rRNA amplicons [21–23]. Dhorne-Pollet et al. [24] performed parallel sequencing of several mitogenomes using an exonuclease depletion step followed by ONT sequencing, though it remains to be determined if this protocol can be scaled up to hundreds of samples [also see 25]. Vossen & Buermans [26] employed a PCR-based indexing approach to sequence a human mitogenome in two slightly overlapping fragments, but did not pool multiple samples together. During revision of this manuscript, Kneubehl et al. [27] introduced an ONT approach to sequence 85 tick mitochondrial genomes using a similar long amplicon approach as we present here. We sought to leverage these new developments to highly multiplex mitogenomes using long-amplicon sequencing. Our methodology, similar to that of Vossen & Buermans [26], involves two overlapping fragments that are asymmetrically indexed.

## Materials and methods

### Laboratory methods

Tissue samples of lizards were collected over the course of several field seasons in Indonesia under appropriate permits as part of a biotic survey of mountains across the island of Sulawesi. Liver tissues were dissected and stored in RNA-later, which were incubated at ambient temperature for 24 to 48 h and then transferred to liquid nitrogen for long-term storage. DNA was extracted from these tissues using either salt precipitation [28 with modifications] or SPRI bead extraction [29]. Resulting DNA precipitates were dissolved in LTE buffer (10 mM Tris, 0.1 mM EDTA pH = 8), quantified using a NanoDrop spectrophotometer, and diluted to a concentration of ~ 35 ng/µl.

**Fig. 1:**
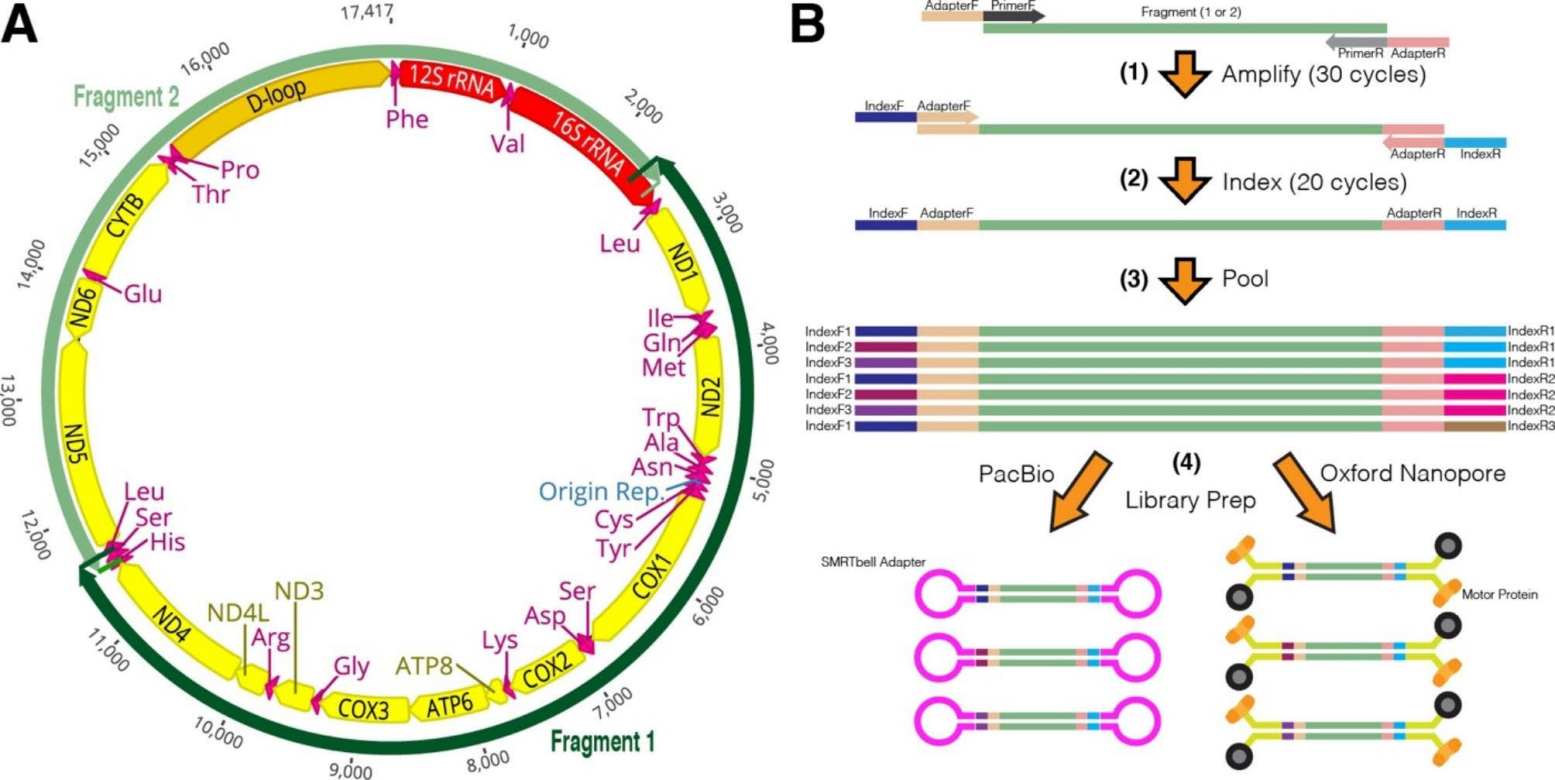
(A) Schematic of an annotated skink mitogenome (*Sphenomorphus incognitus*, MH329292*)* displaying primer binding sites of long-range PCR Fragments 1 and 2 (green outer lines). Note that the fragments narrowly overlap. (B) Overview of lab workflow: (1) first-round PCR with tailed primers targeting primer binding sites and resulting in universal adapters attached to PCR fragments; (2) second-round PCR targeting the universal adapters and resulting in indexed fragments; (3) equimolar pooling of asymmetrically indexed fragments (note that Fragment 1 and 2 of the same sample received an identical index pair); (4) separate library preparation protocols for PacBio and Oxford Nanopore sequencing

We employed a modified two-step PCR approach based on a standard PacBio amplicon sequencing protocol [30] to amplify the mitogenome in two slightly overlapping fragments (see Fig. 1). In brief, the first round of PCR targeted two amplicons per specimen using two distinct reactions each with unique primer pairs. The primers utilized in the first round were designed with a universal adapter tail. The second dual-indexing PCR utilized primers that targeted the universal adapter from the first round of PCR and incorporated asymmetric BUP (Barcoded Universal Primer) tails. In addition to the information below, we provide more detailed laboratory protocols as a supplemental text (Additional file 2).

We conducted PCRs using half-reactions of a long amplification *Taq* polymerase (New England Biolabs (NEB) LongAmp *Taq* DNA Polymerase M0323L), which we chose due to its significantly lower price compared to other long-range polymerases. We note that PCR errors will be more common using this enzyme (its fidelity is 2x better than standard *Taq*) when compared to high-fidelity polymerases (e.g., Phusion Hot Start II which can be 50x better than *Taq*). Given the large fragment size and high number of cycles from two PCR reactions, it is estimated that no amplicons will be exact copies and each will likely contain several errors [31]. Still, given that our goal was to build mitochondrial phylogenetic trees across divergent species these random errors are unlikely to lead to substantial biases given the overwhelming phylogenetic information content available in the long and informative mitochondrial DNA sequences. Other use cases, such as medical applications, may prefer to use high-fidelity polymerases or alternative amplification-free methods for mitogenome sequencing.

We designed PCR primers by aligning mitochondrial genome sequences from GenBank across scincomorph lizards (NCBI nucleotide database search term: “scincomorpha mitochondrion complete genome”) and then using Primer3 v2.3.7 [32] implemented in Geneious R11 [33] with the appropriate universal adapter added as a 5’ extension. We searched for primers with a priming region between 19 and 22 bp in length and otherwise default settings. We confirmed that resulting primers did not form problematic secondary structures using the IDT online primer analysis tool [34] by confirming all structures had Delta G less than − 9. We searched for primers in several conserved regions of 16 S rRNA that we found to be nearly 100% conserved across all Squamates. The 16 S rRNA forward and reverse primers were separated by 99 bp which corresponds to the overlap region between the two fragments. We then looked for possible primers on the opposite side of the mitochondrial genome and chose to target the tRNAs between the ND4 and ND5 genes. Specifically, we designed a new forward primer within tRNA-His in the same area that is targeted by a reverse PCR primer designed to target the ND4 gene for Sanger sequencing [35]. We designed another primer within tRNA-Leu in an area also commonly used to capture the ND4 gene [36]. The tRNA-His and tRNA-Leu primers were separated by 82 bp which corresponds to the opposing overlapping region between the two fragments. The corresponding amplification region from 16 S-F to tLeu-R is approximately 9288 bp and from tHis-F to 16 S-R approximately 8396 bp (varies depending on D-loop length). First-round PCR primers were ordered as HPLC purified and with a 5’ block (NH4–C6) to ensure non-indexed amplicons from the first PCR were not ligated to SMRTbell adapters. Primer sequences are listed in Table 1.

**Table 1:** Primer sequences (5’ to 3’) developed for this study. Universal adapter sequences denoted by lower case letters. Primers were HPLC purified and ordered with a 5’ block (NH4–C6) to ensure first-round products were not ligated to SMRTbell adapters. Position refers to the 3’ end of the primer on the *Sphenomorphus incognitus* mitochondrial genome (Fig. 1) with base 1 referring to tRNA-Phe and continuing through 12 and 16 S rRNA.

Fragment	Position	Primer Name	Primer Sequence
1	2,369	16SF_skink_AF	/5AmMC6/gcagtcgaacatgtagctgactcaggtcacTATCGACAAGAAGGTTTACGAC
1	11,614	tLeuR_skink_AR	/5AmMC6/tggatcacttgtgcaagcatcacatcgtagATCCTTTAAAAGTGARGRGTCY
2	11,351	tHisF_skink_AF	/5AmMC6/gcagtcgaacatgtagctgactcaggtcacGGTTGTGGACCTAAAAATAG
2	2,469	16SR_skink_AR	/5AmMC6/tggatcacttgtgcaagcatcacatcgtagTAGATAGAAACCGACCTGGATT

The first round of PCR using a modified version of the standard NEB LongAmp Taq Polymerase protocol, reducing the volume to a total of 12.5 µl. We amplified each of the two mitochondrial genome fragments separately in two different reactions, each utilizing unique primer pairs designed with a universal adapter tail. The PCR reaction consisted of 2.5 µL of 5x PCR Buffer, 0.75 µL of 10 mM dNTPs, 0.5 µL of LongAmp Taq, 0.5 µL of template DNA at ~ 35 ng/µL, 0.5 µL of each primer at a 10 mM concentration, and 7.625 µL of sterile water. The PCR protocol included an initial denaturing step of 94º C for 30 s, followed by 30 cycles of 94º C for 30 s denaturing, 50º C for 30 s annealing (which can be optimized for each primer pair), 65º C for 10 m extension, and a final extension at 65º C for 10 m. We confirmed the success of the first-round PCR on a 1% agarose gel run with TBE buffer at 180 volts for 25 min alongside a 1 kb ladder, which was stained with ethidium bromide. If a PCR product was not produced in the first attempt, we bead-cleaned the sample using a homemade magnetic bead solution [29] and then attempted again (for a detailed workflow see Additional file 2).

We bead-cleaned the successful PCR products using a 200 µL BenchSmart (Mettler-Toledo-Rainin) and resuspended them in 25 µL distilled water. Although the BenchSmart is a semi-automated 96-well pipettor that speeds up some protocols, all protocols can be adjusted to use a standard multi-channel pipette and plate magnet. We attempted to remove smaller off-target amplicons and carryover primers by bead-cleaning the PCR products with a 0.5x bead ratio, but this was likely unsuccessful because the off-target amplicons were too large in size (as found in later Fragment Analyzer results). We recommend a 0.8x ratio for future users. We confirmed on an agarose gel for a small random subset of samples that the 0.5x bead ratio was sufficient to remove the carryover primers. Based on the results of the first PCR, we categorized the off-target amplicons into three categories: 48% contained clean bands in the targeted range with no evidence of off-target amplification, 26.4% had some faint off-target amplicons, and 16% had strong off-target amplicons.

We conducted the second-round indexing PCR using NEB LongAmp Taq Polymerase on each round-one amplicon separately (though, pooling amplicons from the same sample before indexing is a possible additional cost-saving measure). We ran the indexing PCR under identical conditions as the first-round PCR, except only for 20 cycles rather than 30. A total of 48 BUPs were provided by the UC Davis DNA Technologies and Expression Analysis Laboratory. The universal adapter sequence for the forward primers was (U1) 5’-GCAGTCGAACATGTAGCTGACTCAGGTCAC-3’ and for the reverse primers was (U2) 5’-TGGATCACTTGTGCAAGCATCACATCGTAG-3’. The indexing scheme was asymmetric, with each sample given a unique forward and reverse BUP combination (except in cases outlined below). We organized each of the two PCR fragments per sample with the same index combination though they were indexed in separate reactions and pooled subsequently. The 24 forward BUPs correspond to PacBio barcodes 1001–1024, and the 24 reverse BUPs correspond to barcodes 1025–1048 (see Additional file 3 for index sequences). We ordered an additional 8 forward BUPs (Integrated DNA Technologies), corresponding to 1025–1032. Important Note: this was an error, and we should have chosen 1049–1056 for additional barcodes such that all BUPs would be asymmetric. However, we solved this problem by including the adapter sequence in the bioinformatic demultiplexing step such that even samples with symmetric BUPs were still binned separately based on the combination of BUP plus adapter sequence together. We did not extensively test if this bioinformatic solution created other biases as future users are unlikely to have this same error and it only impacted a total of eight samples.

We bead-cleaned the resulting indexed amplicons as described above and resuspended in 25 µL of water. We quantified the cleaned products using either a NanoDrop ND-1000, or a SpectraDrop 64-well microplate adapter on a Molecular Devices M2 plate reader and then visualized on a 1% Agarose gel to assess the level of off-target amplicons. We categorized samples into 13 groups according to the DNA molarity as well as the degree of off-target amplicons within the sample. These categories included: (1) G — no off-target bands, (2) W — faint off-target bands, (3) S — strong off-target bands, and (4) F — no clear band but adequate concentration (> 5 ng/µL) and included optimistically in the hopes this was due to a gel loading error and some sequence would still be recovered. Within those categories, we used four subcategories of decreasing concentration such that samples could be pooled with a single 10 µl pipette with volumes between 1 and 10 µl. We included a final subpool of samples that appeared to have no distinct band in the indexing PCR but had adequate NanoDrop concentration greater than 2 ng/µl (F). The number of amplicons included in each subpool are as follows (note that each mitogenome has two amplicons): G1—98, G2—222, G3—166, G4—72, W1—109, W2—115, W3—62, W4—20, S1—25, S2—61, S3—86, S4—14, F—109 for a total of 1159 amplicons (see Additional file 3 for sample and subpool information). We visualized the S1 pool on a Fragment Analyzer 50 kb assay (sent out to the UC Berkeley Functional Genomics Laboratory) to determine optimal size-selection cutoffs particularly for samples with a high degree of off-target amplicons. We found about nine off-target amplicons all of which were less than 3,200 bp in length (peaks at 408, 590, 739, 864, 1000, 1176, 1520, 2336, and 3186 bp), so we selected a minimum fragment size of 5 kb that would be used for later BluePippin size-selection. We concentrated the subpools to 5 µg DNA in 30 µl of water using a bead cleanup and submitted to the UC Berkeley Genomics Sequencing Laboratory where they were subjected to BluePippin TM size-selection. The UC Berkeley Genomics Sequencing Laboratory then performed Qubit quantification of the 13 size-selected subpools and pooled them in equimolar proportions. They followed this step with a single library preparation of the combined pool for sequencing on a single PacBio SEQUEL 8 M SMRT Cell.

Given some logistical-constraints and timing of availability of MinION flowcells, we did not sequence the exact same pool of samples on the the Oxford Nanopore comparison. Instead, we took subpools W1, W2, S1, and S2 after the BluePippin prep and pooled those in equimolar proportions with subpools G1, G2, and G3 to create a final pool of 796 amplicons. The pool was prepared for sequencing using the Ligation Sequencing Kit (SQK-LSK110) according to the manufacturer’s protocols. The final library was sequenced on two MinION R9.4 flowcells, one of which was an unused flowcell, and the other was washed with exonuclease after sequencing 2,998,960 bp of ~ 750 bp PCR amplicons for the purposes of plant identifications for the 2021 UC Berkeley Field Genomics course. Each flowcell was cleaned using an additional exonuclease step before loading all remaining available prepared library. Note that since the Nanopore and PacBio sequencing runs did not use an identical pool of samples they are not directly comparable, but rather are used to display the possible utility of each.

### PacBio processing

We collapsed PacBio reads to circular consensus sequences (CCS) using the SMRT-Tools package with default settings with minimum number of complete passes set to 3 to ensure asymmetric barcodes were read. We plotted the distributions of read lengths for both the PacBio (post-CCS) and ONT data using NanoPlot v1.40.0 [37].

We demultiplexed the CCS reads using Lima v2.6.0 (available from https://github.com/pacificbiosciences/barcoding/), with adapter sequences included with the barcode (to deal with the error mentioned above of some sequences with oppositely identical BUPs after flipping Forward/Reverse BUPs). We set the minimum score threshold to 80, and we excluded all CCS sequences less than 7,000 bp or greater than 10,300 bp in length accounting for an estimated fragment size ~ 8700 bp.

We used NGSpeciesID [38] parallelized with GNU Parallel [39] to generate consensus sequences for each of the two fragments per sample. NGSpeciesID uses a reference-free approach to separate reads into bins and assembles consensus sequences using iterative mapping to other reads. The benefit of using NGSpeciesID with these data is that incorrectly demultiplexed reads (if they represent a different species) are placed in a separate consensus sequence cluster and do not affect the output consensus sequence. For most samples, NGSpeciesID generated two consensus sequences, one for each amplicon fragment of the mitogenome. Consensus sequences were called for all NGSpeciesID clusters, even those with just one read. 40 of 676 samples NGSpeciesID produced more than two consensus sequences, with these additional consensus sequences corresponded to low numbers of incorrectly demultiplexed reads. We were able to remove incorrect consensus sequences later as they usually had much lower coverage and often were placed clearly incorrectly in the phylogeny outside of the expected species group or in another genus altogether. We separated NGSpeciedID consensus sequences into Fragment 1 and 2 bins using Geneious by first sorting by length (Fragment 1 ~ 9.3 kb, Fragment 2 ~ 8 kb) and then annotating from *Sphenomorphus incognitus* (MH329292) to visually separate any incorrectly length-binned fragments.

### ONT processing

ONT reads were basecalled using MinKNOW 4.3.20 with Guppy using the Super Accurate Basecaller. The reads were demultiplexed using Minibar v0.21 [21] using default settings and a separate input file specifying only samples that were included in the ONT sequencing pool. Minibar allows one to specify the edit distance, which is the number of bases that differ between the read and the true index sequence. After testing multiple values, we chose an edit distance of 1 which resulted in a good balance of extracting enough reads but reducing off-target reads. Values higher than this could not differentiate between some BUPs, which is a primary reason why ONT-optimized BUPs must be used for ONT-focused experiments. We note that the BUPs we used are optimized for PacBio sequencing chemistry rather than ONT, and therefore we expect performance to be reduced with ONT. If using ONT alone, we recommend longer indexes optimized for reduced error on the ONT platform [see 22]. We discarded outputs with ambiguous specimen identity based on the edit distance. Consensus sequences were assembled using NGSpeciesID as described for the PacBio sequences above.

### Phylogenetic analyses

We tested if analyses of mitogenomes result in better-supported phylogenetic trees compared with single mitochondrial gene trees. This would provide another motivating reason to capture larger genetic regions using this or similar methods to increase phylogenetic information content without increasing costs. To limit bias from missing data and other factors, we limited our analysis to samples with both mitogenome fragments sequenced from the PacBio sequencing dataset and trimmed the full alignment to shorter alignments with the exact same set of tips. We included 266 ingroup taxa with full mitogenomes and 18 scincomorph outgroup mitogenomes from GenBank. Mitogenomes from GenBank included 16 species of Scincidae: *Asymblepharus himalayanus* (NC_058309), *Ateuchosaurus chinensis* (NC_057221), *Isopachys gyldenstolpei* (NC_041147), *Scincella huanrenensis* (NC_030779), *Scincella modesta* (NC_048521), *Scincella vandenburghi* (NC_030776), *Scincella reevesii* (NC_054206), *Sphenomorphus indicus* (NC_045408), *Sphenomorphus incognitus* (NC_041124), *Tropidophorus hangnam* (NC_050664), *Plestiodon chinensis* (NC_029352), *Plestiodon elegans* (NC_024576), *Plestiodon tunganus* (NC_045232), *Plestiodon liui* (MT662111), *Plestiodon egregious* (NC_000888), *Eutropis multifasciata* (MN938934); one of Cordylidae: *Smaug warreni* (NC_005962); and one of Xantusiidae: *Lepidophyma flavimaculatum* (NC_008775). These represent all species with currently available mitogenomes within the 1887 species and 4 families of Scincomorpha [40].

The circular mitogenomes from GenBank needed to be linearized for easy alignment to the remaining sequences. We did so at the Fragment 1–16 S rRNA primer binding site such that the overlapping 99 bp 16 S rRNA region between the Fragment 1 and 2 primer sites was duplicated at the beginning and end of the outgroup sequence alignment. Using the GenBank annotations we manually trimmed out the D-loop as it was too variable to be accurately aligned. For the ingroup alignment, we first created separated Fragment 1 and 2 alignments using MAFFT [41], then transferred annotations in Geneious from the *Sphenomorphus incognitus* mitogenome (MH329292) reference [42]. These preliminary annotations were not curated and only used for trimming alignments. We used these annotations to manually trim out the 82 bp overlapping region between the tRNA-His and tRNA-Leu primers, then concatenated the Fragment 1 and 2 alignments together, removed all sequences that did not have both Fragments represented, and removed the D-loop and duplicated 16 S rRNA region. We then aligned the 266 ingroup sequences to the 18 outgroups using MAFFT, and made iteratively smaller alignments, first to the Fragment 1 region (aligned length 9636 bp), and then to the ND2 gene region (aligned length 1039 bp). Sequence alignments can be accessed in Additional file 5.

We analyzed the resulting three alignments using IQ-TREE v2.1.3 [43], as it allows for automatic model selection and concurrent estimation of multiple support values. To eliminate bias, we did not partition any analysis and allowed IQ-TREE to choose the model automatically using the MFP option. We ran 1000 ultrafast bootstrap (UFBoot) replicates and 1000 approximate likelihood-ratio test (ALRT) replicates for each analysis to measure support. Support values were extracted and compared using the *ape* package [44] in R [45]. We compared the three groups under a one-way ANOVA followed by Tukey’s HSD post-hoc test. Note that these data violate the assumptions of these tests as they are extremely left-skewed and non-normally distributed, but the ANOVA should still be robust to these violations.

## Results

### Pacbio sequencing

**Fig. 2:**
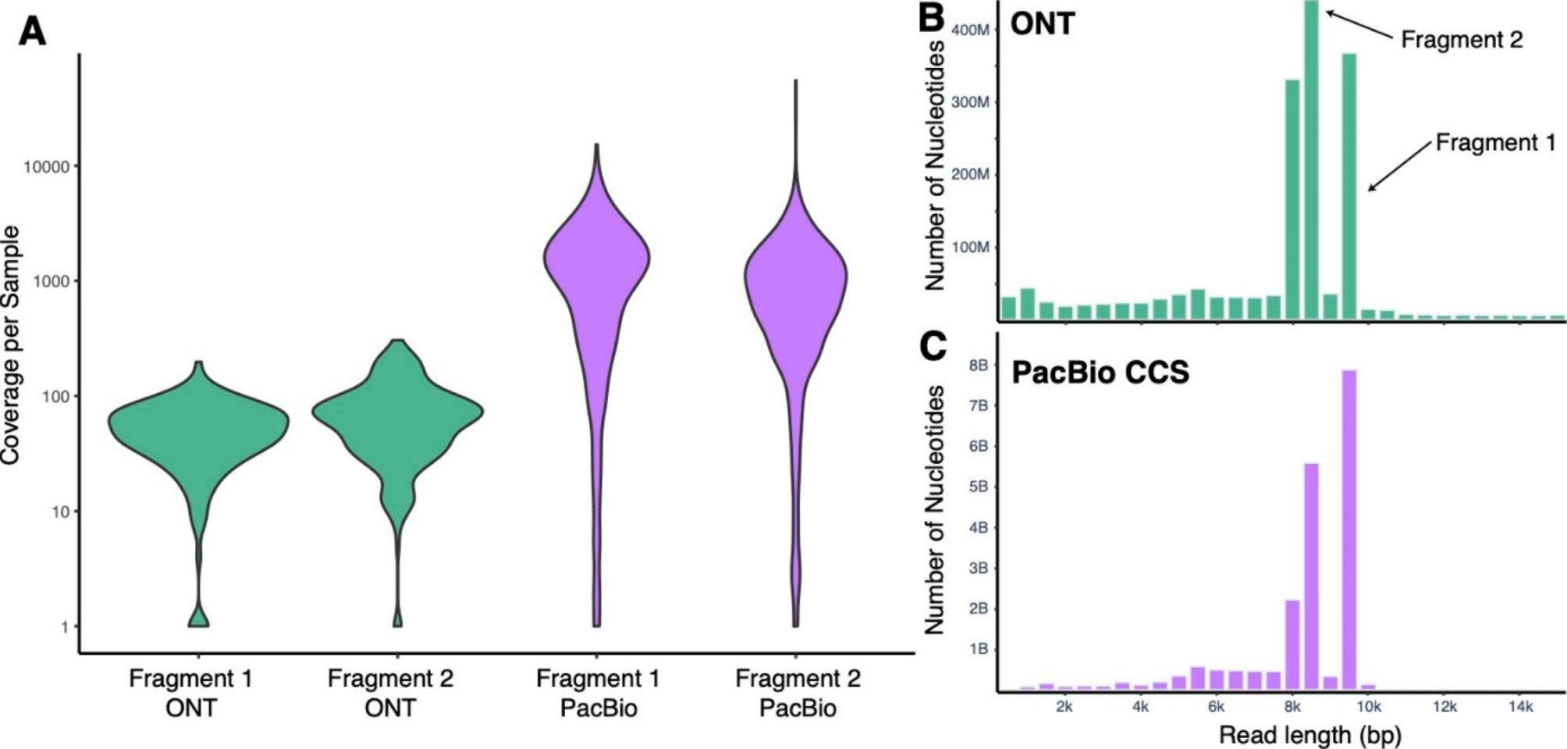
(A) Violin plots displaying coverage per sample across sequencing platforms and amplicon fragments (log transformed for easier visualization). Note that ONT data have lower coverage because we used non-optimized BUPs (see text). PacBio coverage is based on post-CCS reads, so even a coverage of 1 or 2 may be a relatively high-quality sequence. (B and C) Weighted histograms of read lengths for ONT (combined runs) and PacBio CCS reads with Fragment 1 and 2 peaks displayed. Bar height is weighted by read length — the number of reads multiplied by the length of the read

The PacBio SMRT Cell produced 19.93 Gb of sequence data from 7,025,135 polymerase reads with a mean length of 77,100 bp (full read length before circular consensus sequences were called). The subread length distribution was bimodal, with a first peak at 1–2 kb and a second peak around the desired 8–9 kb. The CCS produced 2,802,810 reads (39.9%) passing the filters, with reads lacking full passes and CCS below minimum accuracy accounting for more than 80% of failures. Demultiplexing with Lima produced 1,346,288 reads, with 51% of input reads failing primarily either the length or score thresholds (Fig. 2C).

NGSpeciesID on the PacBio sequencing data resulted in 438 Fragment 1 consensus sequences (of 571 input samples) and 487 (of 588 input) Fragment 2 consensus sequences. The consensus sequences had a mean coverage of 1380x for Fragment 1 and 1420x for Fragment 2 (see Table 2; Fig. 2A). Coverage among subpools varied widely (see Additional file 6 Fig. S2). The F subpool containing samples with no visible bands accounted for 36% of failures (see Additional file 4 for sample-by-sample sequencing results), though 19 of the 109 samples in this subpool did recover a consensus sequence. Given this, we calculated failure rates with and without accounting for samples in the F pool. Subpools with strong off-target amplicons (S1–4) had a higher failure rate of 31% compared to the other subpools (12.2% failed in G1–4 and 10.5% failed in W1–4).

**Table 2:** Summary of coverage per amplicon or per sample produced by NGSpeciesID. Note that reduced recovery of ONT samples due to non-optimized BUPs. We also calculated the percent recovery after removing samples that were in the F subpool as these were only included optimistically despite having no clear post-PCR gel band (see text for details)

Group	Samples/ Fragments Input	Samples/ Fragments Recovered	Recovery After Accounting for F pool	Mean Coverage	SD	Min	Max
ONT Fragment 1	335	142 (42.3%)		51.5x	30.4x	1x	197x
ONT Fragment 2	461	224 (48.5%)		79.8x	61.9x	1x	305x
ONT Fragment 1 and 2	549	116 (NA)^1^					
ONT Fragment 1 or 2	549	250 (45.5%)					
PacBio Fragment 1	571	438 (76.7%)	83.7%	13,80.3x	1,500.1x	1x	15,261x
PacBio Fragment 2	588	487 (82.8%)	89.0%	1,420.2x	4,147.1x	1x	54,970x
PacBio Fragment 1 and 2	677	266 (39.2%)	44.5%				
PacBio Fragment 1 or 2	677	649 (95.9%)	97.9%				

^1^Not comparable because the ONT pool did not contain equal quantities of Fragments 1 and 2 for each sample

### ONT sequencing

The combined ONT sequencing runs produced 1.81 Gb of passing data from 397,885 reads with a mean length of 4,538 bp (sd = 4,019 bp) (Fig. 2B). The majority of these data were from the unused flowcell, which produced 1.57 Gb of data from 297,954 reads with a mean length of 5,269 bp (sd = 3,921). The remainder is from the partially-used flowcell which produced only 0.24 Gb of data from 99,931 reads with a mean length of 2,360 bp (sd = 3,484). Note that there were likely some carryover small amplicons on the used flowcell (see Additional file 6 Fig. S1 for distribution of reads from each flowcell displayed separately). These carryover amplicons were filtered out during the demultiplexing step with our read-length thresholds. The consensus sequences had a mean coverage of 52x for Fragment 1 and 80x for Fragment 2 (Table 2; Fig. 2A).

### Phylogenetic analyses

**Fig. 3:**
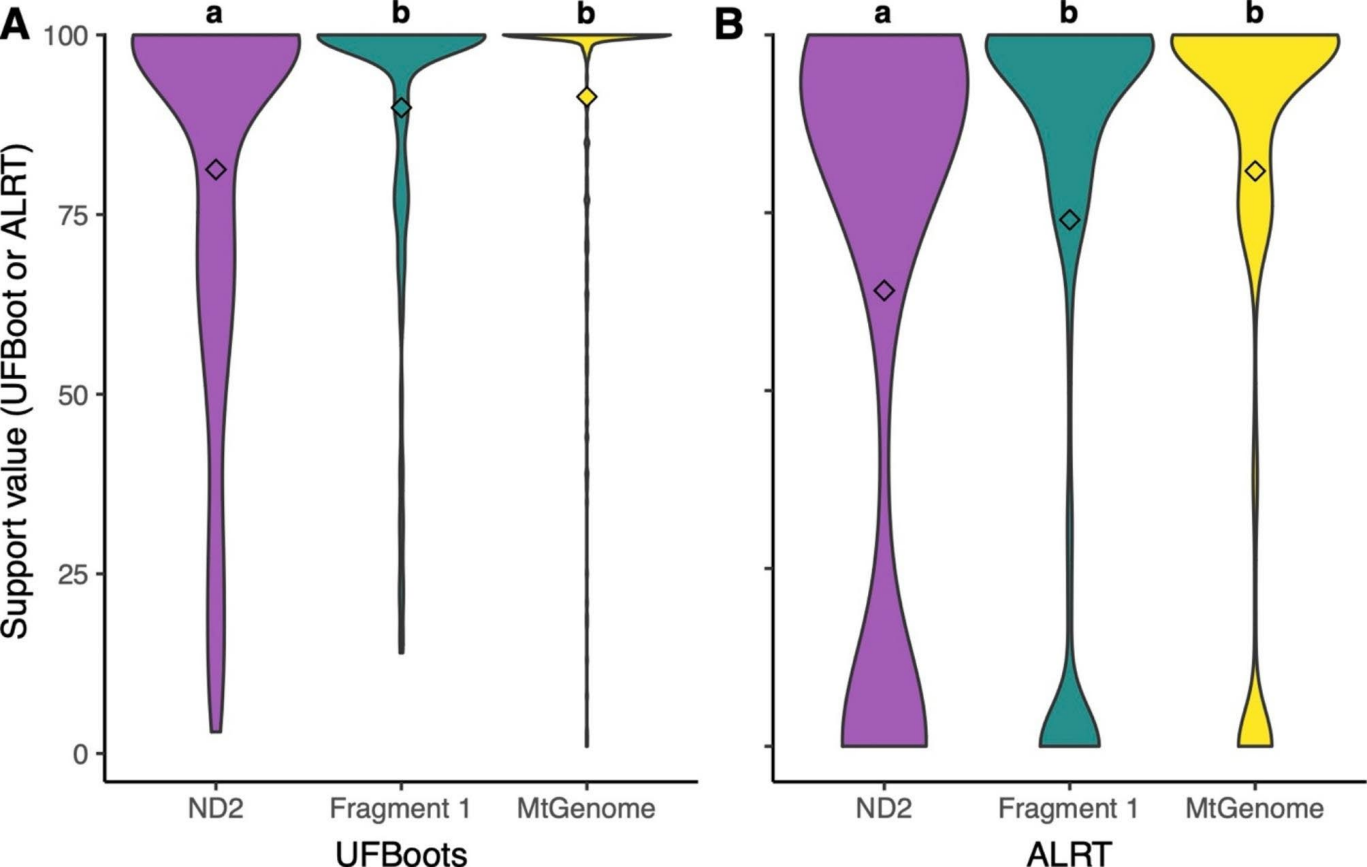
Violin-plots comparing tree support values for different sized alignments with means denoted by a diamond. We compare the ND2 gene alone (1,039 bp), Fragment 1 (9,636 bp), and the entire mitochondrial genome excluding the D-loop (16,654 bp) for (A) ultrafast bootstraps (UFboots) and (B) approximate likelihood-ratio tests (ALRT) implemented in IQ-TREE. Violins have equal widths for easier visualization of the distributions. Groups with significantly different means by Tukey’s HSD test are labeled with different letters above the plots

Both UFBoot and ALRT values for ND2 (~ 1 kb) alone were significantly lower than those for the entirety of Fragment 1 (~ 9 kb) or the entire mitogenome excluding the D-loop (~ 16 kb) (Fig. 3). Mean UFBoot values were 81.3 (SD = 27.1) for ND2, 89.9 (SD = 20.0) for Fragment 1, and 91.4 (SD = 20.0) for the full mitogenome. Mean ALRT values were 64.1 (SD = 41.6) for ND2, 74.0 (SD = 37.9) for Fragment 1, and 80.9 (SD = 32.9) for the full mitogenome. ANOVA for both UFBoot and ALRT values were significant (p = 8.49e-7 and p = 4.04e-6, respectively). Tukey’s HSD test found Fragment 1 and the full mitogenome had significantly higher support than the ND2 gene alone for both UFBoot and ALRT values. Fragment 1 and the full mitogenome were not significantly different from one another for either UFBoot or ALRT values. Maximum likelihood trees for each analysis can be found in Additional file 6 (Figs. S3–5).

## Discussion

Our approach successfully and efficiently recovered hundreds of mitogenomes. We had a high rate of recovery of 95.9% of input samples with one of the two fragments in the PacBio run; however, substantially fewer samples (39.2%) recovered both Fragment 1 and Fragment 2 together. 36% of amplicons that failed to sequence were in the F subpool that were optimistically included despite having no clear band on the post-PCR gel but adequate quantified nucleic acid concentration. After accounting for these failures, our recovery rate was 97.9% of samples with one of two fragments and 44.5% with both fragments. Other failures were associated with samples with strong off-target amplicons, indicating that optimizations of PCR reaction conditions to reduce off-target amplicons (e.g., touchdown protocols that lower the annealing temperature as the cycles progress) may be worth exploring. Based on our experience, we have several recommendations for future users continue to use and develop this or similar protocols.

First, it is important to note that since our protocol is based on PCR amplification, the method will have varying success at different evolutionary depths depending on the primer regions chosen. With primer design, one must balance reducing off-target amplicons with broad applicability across multiple scales of life. For example, a researcher working on a single species may select primers with no degeneracy and in regions that would otherwise be too variable to amplify across the family. We chose ~ 20 bp primers as we have had success with primers of this size in past Sanger experiments. We expect we would have been more successful sequencing both fragments across more of our samples if we had been working at a shallower evolutionary scale and could have used more specific primers (i.e., within a lizard genus versus across a family).

Another trade-off in project design is whether one should target one long fragment (~ 17 kb) or two shorter fragments (~ 9 kb each). The benefits of using a single long fragment are that one can use more conserved primer binding sites such as highly conserved regions of 16 S rRNA [see 46] and that it reduces the lab work by almost half [47]. The downsides are that ≥ 15 kb amplifications can be finicky, and they are likely to receive reduced coverage since more of the sequence reads will fall short of the full length and will not demultiplex using an asymmetric index scheme. Given that we recovered substantially higher than required per-sample coverage on the PacBio sequencing run (and likely would have also with ONT if we used ONT-optimized indexes and with higher throughput), we don’t expect the latter issue to be a major problem if relatively even pooling is achieved. We chose to target two overlapping fragments mainly to ensure adequate coverage with our indexing scheme and less-troublesome PCR; however, given the large number of samples that did not recover both fragments either because they would not amplify in the first place or did not sequence, we may have been better off with a single long amplicon [as in 46]. Based on our experience, our recommendation to others seeking to use this method is to use two fragments when working with lower quality tissue samples or at shallow phylogenetic scales (i.e. within a genus or similar) to maximize number of reads with sufficient length, and to use a single fragment at deeper scales (i.e. family level or above) and with high quality tissue samples. Using more than two fragments would likely have the downside of a higher proportion of samples missing at least one fragment.

Another important consideration is whether the whole mitochondrial genome sequence is truly necessary to answer the research question at hand. If phylogenetic resolution is the main interest, we found that a single 9 kb region already led to a much better supported gene tree when compared to a single mitochondrial gene. This is expected, and consistent with substantial work showing that longer alignments produce more robust gene trees from increased information content [48, 49], though our results suggest these gains diminish at increasing lengths. On the other hand, if one is working within a single species then the D-loop may be of higher importance as it may contain most of the phylogenetic information (as for humans). In the focal lizard radiation we studied here we do find slight improvement in average support values (not statistically significant) with the full mitochondrial genome compared to Fragment 1 alone (Fig. 3); however, this slight benefit may not be worth the increased workload and cost. Furthermore, the D-loop region was not easily aligned and had to be clipped from our final alignment. It may be better to sequence a few long nuclear amplicons, such as nuclear rRNA [21] or a few long, rapidly evolving, and highly informative nuclear exons [50] for a multi-locus dataset with ample phylogenetic information content. It is possible that several of these amplicons could be multiplexed under the same PCR reaction mix, but one runs the risk of amplifying a greater proportion of off-target regions with additional primer pairs. However, if the target regions are comparable in size, then one could remove off-target amplicons with gel excision or BluePippin size selection as we did here. If more than two amplicons per-sample are targeted, substantial cost reductions are possible if one pools the fragments after the first PCR step resulting in a single indexing PCR per sample. It would be possible to utilize this modification even for the two-fragment experimental design presented in this manuscript, but we chose to pool after indexing as we expected there to be a higher risk of uneven pooling given only a slight cost-benefit with just two fragments.

Based on an assumption of 2.8 million CCS reads as we recovered here, we estimate that with even coverage across samples we could theoretically multiplex as many as 140,000 mitochondrial genomes (two-fragment) on a single SMRT cell to achieve a mean coverage of 10x. However, given that we took shortcuts in pooling samples such that resulting coverage varied widely (see Additional file 6 Fig S2), more poorly represented samples in the sequencing pool may not generate any sequences. We therefore recommend estimating throughput based on a mean coverage of 200x, which would still allow for 3,000–7,000 mitochondrial genomes on a single SMRT cell (see Additional files 1 and 4 for cost considerations).

It remains to be seen how well this method will perform on the ONT platform once more appropriate BUPs are utilized and with additional sequence data, but we expect it to work well based on the moderate output we recovered here. If so, it will open-up even more cost-efficient mitogenome sequencing in field settings and areas with limited laboratory resources [6]. One can expect to recover 4–20 Gb of data on a single MinION flowcell [51–54] which is much higher than the 1.6 Gb we recovered here on the fresh MinION flowcell, and generally amplicons are expected to yield higher throughput than genomic DNA. We may have recovered higher throughput on our ONT sequencing if we had prepared more input library for sequencing and used it entirely on the fresh flowcell rather than splitting it between the two as we ran out of library even though we still had active pores. We estimate that if a MinION flowcell can yield 1 million reads for 8–10 kb amplicons that one could multiplex 2,500 samples based on a mean coverage of 200x.

**Declarations**.

## Electronic supplementary material

Below is the link to the electronic supplementary material.

**Additional file 1:** Cost considerations.

**Additional file 2:** MtGenome for the cost of a gene: Long-amplicon PacBio and Nanopore sequencing of hundreds of full mitochondrial genomes.

**Additional file 3:** Table S1. Samples included in PacBio sequencing analysis and their respective PCR success, pool category, and whether they were sequenced and the resulting coverage.

**Additional file 4**

**Additional file 5**

**Additional file 6: Supplemental Fig. 1.** Weighted histograms displaying read lengths of each ONT flowcell. **Fig. S2.** Violin plots displaying PacBio sequence coverage for consensus sequences of samples from each subpool. **Fig. S3.** Maximum likelihood phylogeny from IQTREE for the ND2 gene with bootstrap node support. **Fig. S4.** Maximum likelihood phylogeny from IQTREE for the Fragment 1 alignment with bootstrap node support. **Fig. S5.** Maximum likelihood phylogeny from IQTREE for the full mitochondrial genome alignment with bootstrap node support.

## Data Availability

Sample information and subpool information can be found in Additional file 3. Sequence alignments used for the comparison of gene trees can be downloaded in Additional file 5 and maximum likelihood trees in Additional file 6. The demultiplexed raw PacBio CCS reads can be accessed from the NCBI sequence read archive (PRJNA945206, SAMN33774969–5644). Other data will be provided on request.

## Declarations

Fieldwork in Indonesia was carried out under research permits issued by RISTEKDIKTI (233/SIP/FRP/SM/VI/2013) and UC Berkeley IACUC protocol #R279.

*Consent for publication*.
