# Supplementary material for: Highly-multiplexed and efficient long-amplicon PacBio and Nanopore sequencing of hundreds of full mitochondrial genomes
Source: BMC Genomics. 2023 May 2;24:229. doi: 10.1186/s12864-023-09277-6 (PMC10155392; doi:10.1186/s12864-023-09277-6)
Supplement: Supplementary file 1 — Additional file 1: Cost considerations. [file 12864_2023_9277_MOESM1_ESM.pdf]

Cost Considerations

Sanger sequencing has been long used to screen mtDNA markers, but, because of its maximum length of 500–1000 bp per read, the cost and workload scales up quickly if longer gene regions are desired [1]. The advent of Illumina sequencing provided a mechanism for researchers to quickly sequence mitogenomes, but per-sample costs also increase linearly as most methods utilize a ligation-based library preparation step that carries a fixed per-sample cost that is often ~$40 or more and in labs with ample resources can be reduced to $15–20. With specialized modifications to Nextera Flex, library-costs can be reduced to $7.22 for large numbers (4000–5000) of samples [2], with thousands of dollars of additional sequencing costs still required for these large-scale projects.

In Additional file 3, we estimated costs for similar long-amplicon sequencing projects (post- DNA extraction) based on lab costs for UC researchers. Lab costs are based on those at the Museum of Vertebrate Zoology Evolutionary Genetics Lab and sequencing costs at the UC Davis DNA Technologies & Expression Analysis Core under the UC rate [3]. Using Table S1, we estimate the total cost for a similar experiment at $4,555 or $6.73 for each mitogenome for 677 samples. We note that costs may differ substantially depending on the available resources at a particular laboratory, especially for sequencing and BUPs. We recommend interested users to carefully estimate costs based on their own lab resources by comparing to the supplemental cost table (Additional file 3).

These costs do not necessarily include factors like optimization of PCR reactions, repeating failed PCRs, mistakes, etc. Due to those factors, our real-world costs for this project were slightly higher — $8.27 per mitogenome — inclusive of two rounds of PCR and SPRI bead purification, DNA assessment with agarose gel and spectrophotometer, pooling and size-selection, and one PacBio SMRT cell. As a comparison though, the UC Berkeley DNA Sequencing core charges $7.60 to obtain double-stranded Sanger sequencing data from a single cleaned PCR product up to 1000 bp. So, for a small amount more money and effort we were able to obtain the whole mitogenome instead of a single locus.

The main cost-saving measures we employed were halved reaction sizes, the low-cost long amplicon DNA polymerase, home-made SPRI bead solution, and spectrophotometer rather than fluorescence quantification.

Our approach was successful in that it resulted in hundreds of mitogenomes at a very low per-sample cost as low as ~$7, which is comparable to the cost of a Sanger sequencing a single mitochondrial gene through a core facility [4]. On the PacBio platform, we estimate that at 3,000 samples one could reach a per-sample mitogenome cost of $4.54 (if low-cost BUPs are not available to a researcher, costs will differ substantially from these estimates), and significant cost reductions are possible if one eliminates cleanup steps by PCR optimization or multiplex PCR (see Additional file 4).

ONT costs for a similar project were estimated the same as above, but under the assumption that one would need to purchase all the BUPs for the desired number of samples at $25 per BUP. MinION flowcell costs were estimated based on the cost of a starter pack. See Additional file 4 for cost estimations. We estimate that if a MinION flowcell can yield 1 million reads for 8–10kb amplicons that one could multiplex 2,500 samples based on a mean coverage of 200x, leading to an estimated cost of $3.00 per mitogenome. After an initial investment in BUPs on either platform, the per-sample costs could be reduced substantially as they are reused for subsequent experiments.

References

1. França LTC, Carrilho E, Kist TBL. A review of DNA sequencing techniques. Q Rev Biophys. 2002;35:169–200.
2. Gaio D, Anantanawat K, To J, Liu M, Monahan L, Darling AE. Hackflex: Low-cost, high-throughput, Illumina Nextera Flex library construction. Microb Genomics. 2022;8:000744.
3. UC Davis Genome Center Rates. https://dnatech.genomecenter.ucdavis.edu/uc-prices/; Accessed 3 Jun 2022.
4. Hebert PDN, Braukmann TWA, Janzen DH, Prosser SWJ, Ratnasingham S, DeWaard JR, et al. A Sequel to Sanger: Amplicon sequencing that scales. BMC Genomics. 2018;19:1–25.
