# Supplementary material for: Highly-multiplexed and efficient long-amplicon PacBio and Nanopore sequencing of hundreds of full mitochondrial genomes
Source: BMC Genomics. 2023 May 2;24:229. doi: 10.1186/s12864-023-09277-6 (PMC10155392; doi:10.1186/s12864-023-09277-6)
Supplement: Supplementary file 2 — Additional file 2: MtGenome for the cost of a gene: Long-amplicon PacBio and Nanopore sequencing of hundreds of full mitochondrial genomes. [file 12864_2023_9277_MOESM2_ESM.pdf]

Supplementary Text

MtGenome for the cost of a gene: Long-amplicon PacBio and Nanopore sequencing of hundreds of full mitochondrial genomes

Long Amplicon Sequencing Protocol

Overview

1. DNA Extraction
2. Optimize PCR reaction
3. First Round PCR
4. Gel electrophoresis
5. Bead cleanup
6. DNA quantification
7. Second Round PCR
8. Bead Cleanup
9. Gel electrophoresis
10. DNA quantification
11. Pool equimolarly into subpools based on concentration and proportion off-target
12. BluePippin on subpools if needed
13. Qubit on subpools, equimolar pooling
14. SMRT Library prep and sequencing

DNA Extraction

Method of your choice.

Designing Primers Tailed with Universal Adapter

1. HPLC purified.
2. Add a 5' block (5' NH4-C6) so that first-round PCR amplicons are not ligated to the SMRTbell adapters during SMRT library prep.

Example: /5AmMC6/gcagtcgaacatgtagctgactcaggtcacPRIMERSEQUENCE

Note: M13 adapters may be used in place of the adapter sequence but the Barcoded Universal Primers for the second round PCR must match these M13 adapters.

Optimize PCR reaction

Use a gradient PCR to determine appropriate annealing temperature for primers with universal adapters. Run PCR under the following conditions for half reaction. We chose a gradient of 48.8–65.0º C for the annealing temperature.

**Reagent**	**µL**
diH2O	7.625
5x Buffer	2.5
dNTPs	0.375
Primer F	0.5
Primer R	0.5
LongAmp Taq	0.5
DNA	0.25

**Cycles**	**Temp (ºC)**	**Time**
1x	94	30s
30x	94	30s
	GRADIENT	30s
	65	10m
1x	65	10m
1x	4	inf

Note 1: It may be worth testing a touchdown protocol to remove more off-target PCR.
Note 2: If you can optimize this step to the point where you have no remaining primer when visualized on a gel you may be able to skip the first bead cleanup step.

First Round PCR

Run a PCR under the following conditions

Reagent	µL
diH2O	7.625
5x Buffer	2.5
dNTPs	0.375
Primer F	0.5
Primer R	0.5
LongAmp Taq	0.5
DNA	0.25

Cycles	Temp (ºC)	Time
1x	94	30s
30x	94	30s
	ANNEAL*	30s
	65	10m
1x	65	10m
1x	4	inf

*Substitute with Annealing temperature determined during optimization step.

Note: 10 minute extension time was chosen for the NEB polymerase rate of 1 kb/min and fragment size over 9 kb. Adjust accordingly for different fragment sizes and polymerases as needed.

Gel Electrophoresis

Confirm PCR products on a gel and repeat as necessary. Of the 12.5 µL reaction volume we used 2.5 µL for visualization. We ran 8–9 kb amplicons for 45 minutes at 150 volts.

Bead Cleanup

Clean-up excess PCR reagents and small off-target amplicons using a homemade SPRI bead solution, cleanup the samples.

1. Add 5 µL (0.5x bead ratio) of SPRI bead solution to each well and tip-mix or vortex, incubate 5–15 minutes. (Note: vortexing should be avoided for very long amplicons)
2. Add plate to magnet, wait 5 minutes for solution to bcome clear
3. Remove supernatant without disturbing beads.
4. Add 180 µL 80% EtOH to each well, wait 60 seconds, remove EtOH
5. Repeat step 4 a second time, then wait ~1–2 mins until all EtOH has just evaporated
6. Add 25 µL Elution Buffer or Water, mix thoroughly to resuspend beads
7. Return plate to magnet, wait 2 minutes for solution to clear
8. Transfer supernatant to a fresh plate.

Note: If you are amplifying smaller amplicons you may need to adjust the bead ratio.

DNA Quantification

Use a nanodrop or platereader to quantify each sample. Dilute high concentration samples to ~10 ng/µL

Note: This step can probably be skipped if the researcher does not have access to a rapid platereader quantification method. Dilutions can also be conducted based on gel intensity and a few NanoDrop values.

Second Round Indexing PCR

Using the Barcoded Universal Primers, run the indexing PCR for 20 cycles under the same conditions as the first.

Reagent	µL
diH2O	7.625
5x Buffer	2.5
dNTPs	0.375
Primer F	0.5
Primer R	0.5
LongAmp Taq	0.5
DNA	0.25

Cycles	Temp (ºC)	Time
1x	94	30s
20x	94	30s
	ANNEAL*	30s
	65	10m
1x	65	10m
1x	4	inf

Note: This PCR could be optimized to avoid the following cleanup
Note 2: If you have less than 500 samples, we recommend using full reaction volumes for this step so that you are sure to have enough DNA for the sequencing (see last step for more details).

Bead Cleanup

Follow the same SPRI bead cleanup protocol as the first-round PCR.

1. Add 5 µL of bead solution to each well and tip-mix or vortex, incubate 5–15 minutes. (Note: vortexing should be avoided for very long amplicons)
2. Add plate to magnet, wait 5 minutes for solution to bcome clear
3. Remove supernatant without disturbing beads.
4. Add 180 µL 80% EtOH to each well, wait 60 seconds, remove EtOH
5. Repeat step 4 a second time, then wait ~1–2 mins until all EtOH has just evaporated
6. Add 25 µL Elution Buffer or Water, mix thoroughly to resuspend beads
7. Return plate to magnet, wait 2 minutes for solution to clear
8. Transfer supernatant to a fresh plate.

Gel Electrophoresis

This time, run the gel electrophoresis after the cleanup so that you can visualize the proportion of off-target fragments remaining.

DNA Quantification

Use a nanodrop or platereader to quantify each sample. Dilute high concentration samples to ~10 ng/µL

Note: Pooling can also be conducted based on gel intensity.

Pooling

Categorize samples into bins based on their concentration and the degree of off-target amplicons. Select categories such that all the samples within the category can be pooled equimolarly while pipetting with the same 10 μL pipette with values between 1.0–10.0 μL to avoid pipetting error. You may need to dilute the highest concentration samples to a lower working concentration. You may need to pipette the lowest concentration samples twice if you are low on DNA.

Note: You may need as much as 2 μg of total DNA for the final pool, and you can estimate BluePippin to have ~35% recovery. Use these values to confirm when estimating total DNA weight that you will have enough product for the sequencing facility.

Submit Samples for Prep and Sequencing

Submit to sequencing facility. Order BluePippin size selection on subpools if needed. Pool final DNA based on Qubit values.
