# Supplementary material for: Highly-multiplexed and efficient long-amplicon PacBio and Nanopore sequencing of hundreds of full mitochondrial genomes
Source: BMC Genomics. 2023 May 2;24:229. doi: 10.1186/s12864-023-09277-6 (PMC10155392; doi:10.1186/s12864-023-09277-6)
Supplement: Supplementary file 6 — Additional file 6: Supplemental Fig. 1. Weighted histograms displaying read lengths of each ONT flowcell. Fig. S2. Violin plots displaying PacBio sequence coverage for consensus sequences of samples from each subpool. Fig. S3. Maximum likelihood phylogeny from IQTREE for the ND2 gene with bootstrap node support. Fig. S4. Maximum likelihood phylogeny from IQTREE for the Fragment 1 alignment with bootstrap node support. Fig. S5. Maximum likelihood phylogeny from IQTREE for the full mitochondrial genome alignment with bootstrap node support. [file 12864_2023_9277_MOESM6_ESM.pdf]

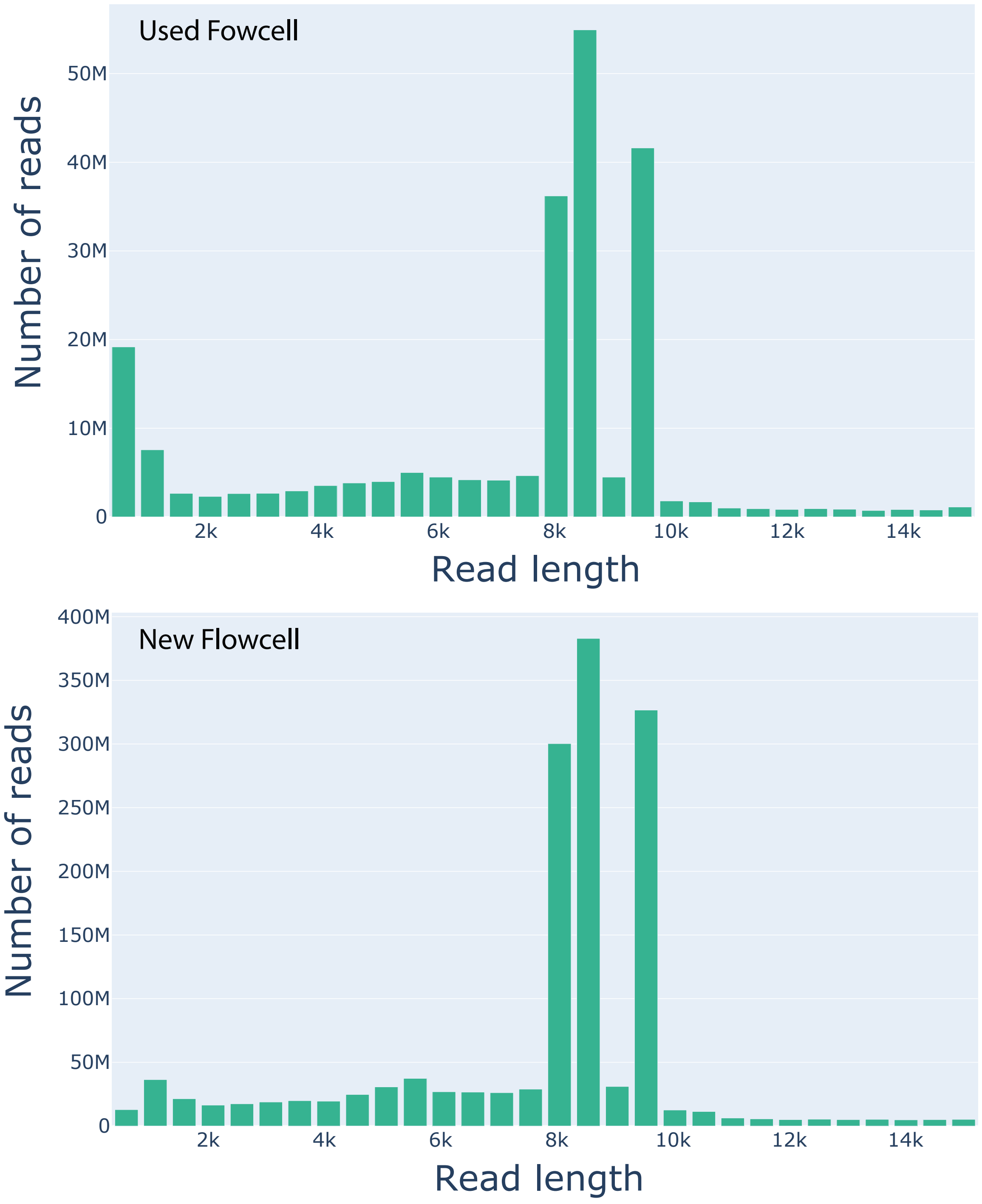

Fig. S1. Weighted histograms displaying read lengths of each ONT flowcell. The used flowcell (top) has short carryover amplicons from a barcoding project.

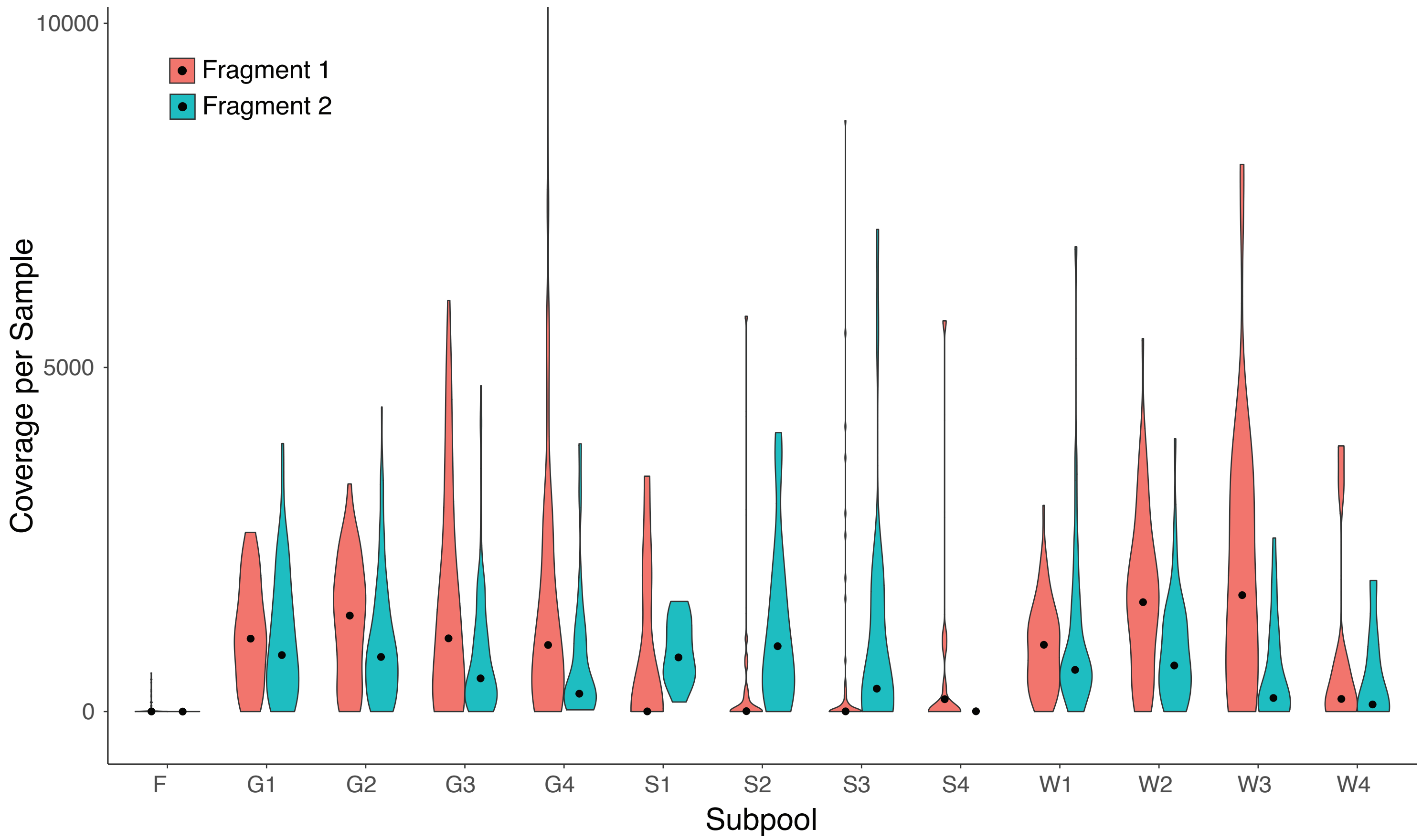

Fig. S2. Violin plots dispalying PacBio sequence coverage for consensus sequences of samples from each subpool. Each subpool is separated into fragment 1 (red) and fragment 2 (green) categories to show variation in coverage. Dots indicate the median coverage within each violin.

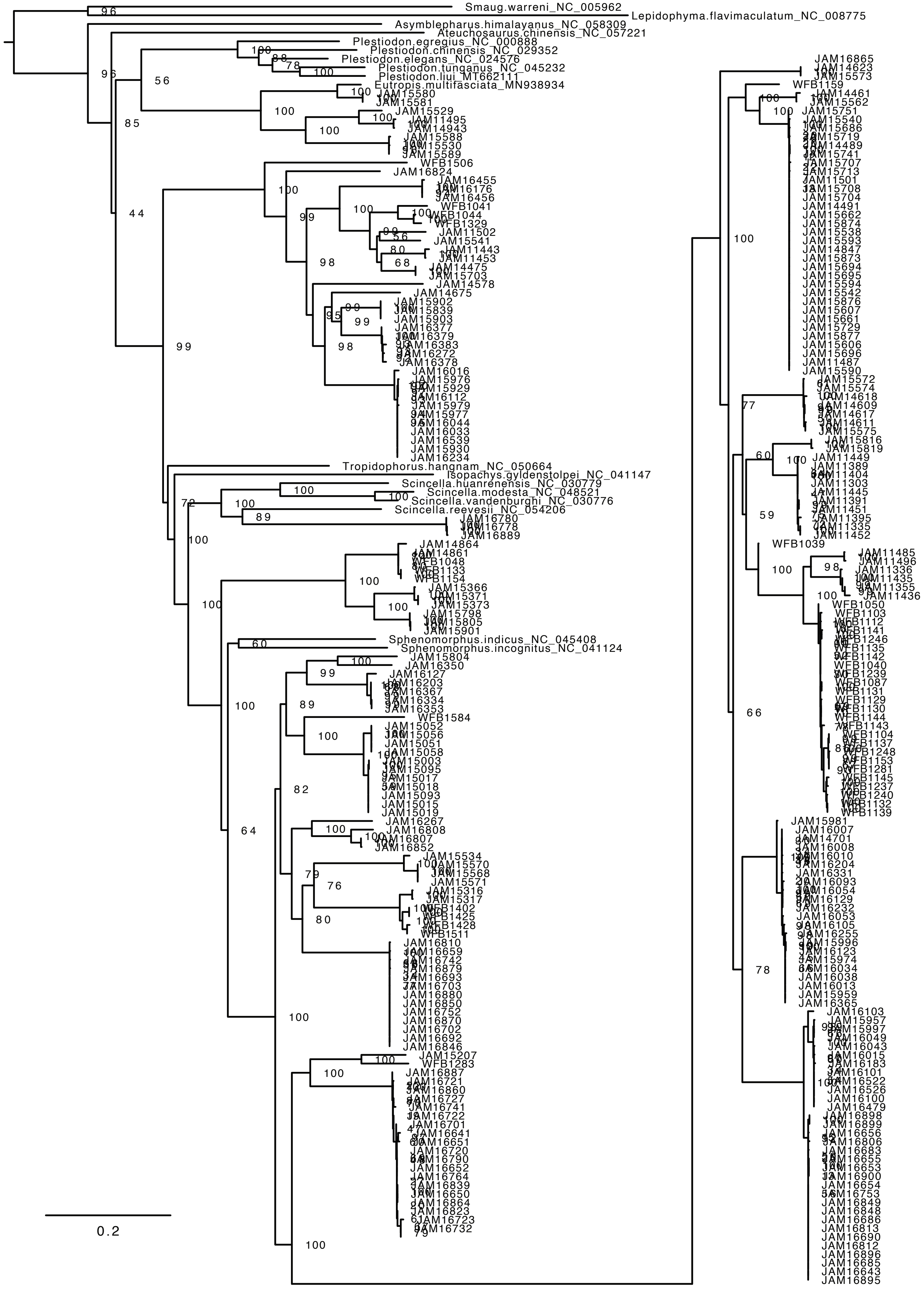

Fig. S3. Maximum likelihood phylogeny from IQTREE for the ND2 gene with bootstrap node support.

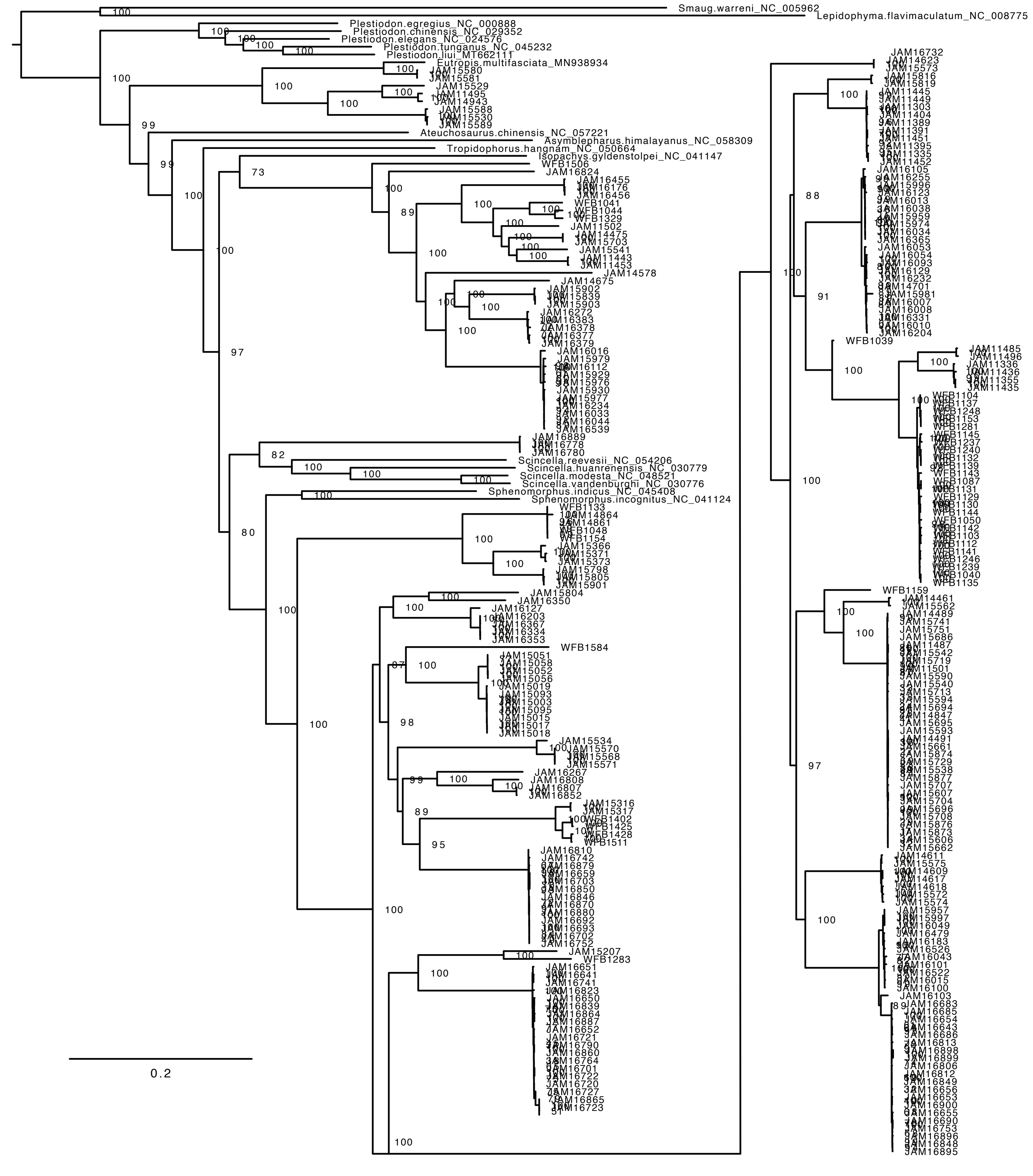

Fig. S4. Maximum likelihood phylogeny from IQTREE for the Fragment 1 alignment with bootstrap node support.

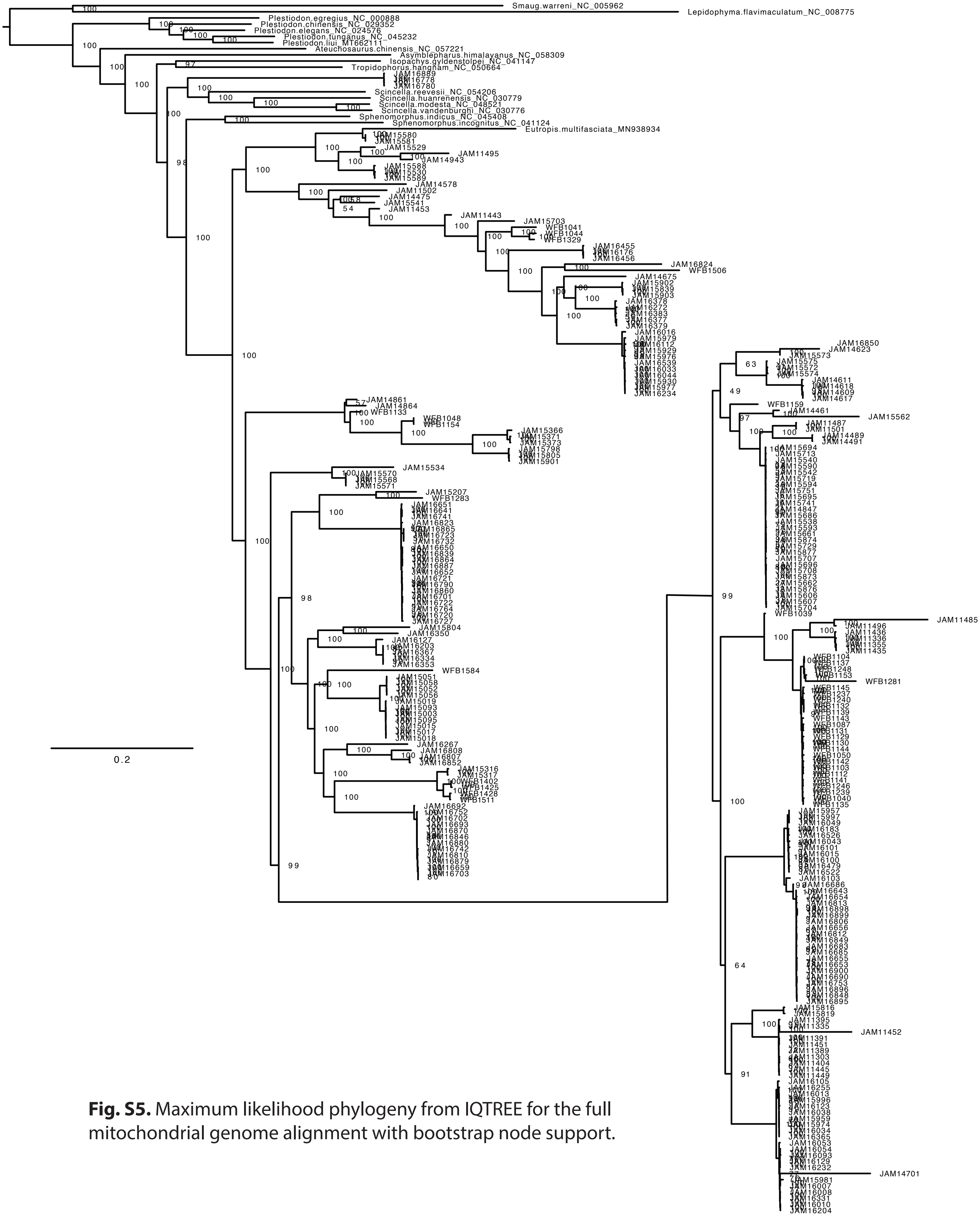

Fig. S5. Maximum likelihood phylogeny from IQTREE for the full mitochondrial genome alignment with bootstrap node support.
